# Supplementary material for: Mechanisms governing the pioneering and redistribution capabilities of the non-classical pioneer PU.1
Source: Nat Commun. 2020 Jan 21;11:402. doi: 10.1038/s41467-019-13960-2 (PMC6972792; doi:10.1038/s41467-019-13960-2)
Supplement: Supplementary file 7 — Source data [file 41467_2019_13960_MOESM7_ESM.zip › Source_Data/FigureS2/FigureS2B_MotifScanOutput/KmeansCluster11/homerResults/motif7.similar.html]

motif7

## Information for motif7

A
T
G
C
G
A
T
C
C
G
A
T
A
G
T
C
A
G
T
C
A
G
T
C
T
G
A
C
T
G
A
C
A
G
C
T
  
Reverse Opposite:  

T
C
G
A
A
C
T
G
A
C
T
G
C
T
A
G
A
C
T
G
C
T
A
G
G
C
T
A
C
T
A
G
T
A
C
G
  

|  |  |
| --- | --- |
| p-value: | 1e-62 |
| log p-value: | -1.428e+02 |
| Information Content per bp: | 1.723 |
| Number of Target Sequences with motif | 1021.0 |
| Percentage of Target Sequences with motif | 31.43% |
| Number of Background Sequences with motif | 8453.8 |
| Percentage of Background Sequences with motif | 18.70% |
| Average Position of motif in Targets | 102.3 +/- 54.4bp |
| Average Position of motif in Background | 99.4 +/- 66.9bp |
| Strand Bias (log2 ratio + to - strand density) | -0.0 |
| Multiplicity (# of sites on avg that occur together) | 1.24 |
| Motif File: | file (matrix) reverse opposite |

### Similar de novo motifs found

|  |  |  |  |  |  |  |  |
| --- | --- | --- | --- | --- | --- | --- | --- |
| Rank | Match Score | Redundant Motif | P-value | log P-value | % of Targets | % of Background | Motif file |
| 1 | 0.940 | A G T C A C G T A G T C A T G C A G T C A G T C A T G C T G A C | 1e-58 | -133.973453 | 32.99% | 20.37% | motif file (matrix) |
| 2 | 0.901 | G A T C A G T C G A T C A T G C A G T C G A T C A G T C | 1e-35 | -81.232496 | 26.10% | 17.02% | motif file (matrix) |
| 3 | 0.880 | C G A T C T A G C A T G C T A G A C T G C T A G G T C A A C T G T C A G T C G A | 1e-33 | -77.393288 | 11.85% | 5.91% | motif file (matrix) |
| 4 | 0.793 | A T G C A G T C A C G T G T A C A G T C G T A C G T A C A G T C C G T A A T G C T C G A T A G C | 1e-20 | -46.690495 | 2.19% | 0.51% | motif file (matrix) |
